# Glucose-6-phosphate dehydrogenase variants modify 3D genomic organization to suppress maladaptive gene expression and vascular disease

**DOI:** 10.1016/j.jbc.2026.113204

**Published:** 2026-05-27

**Authors:** Christina Signoretti, Shun Matsumura, Melinee D’silva, Comfort Williams, Brenden Marshall, Samuel Fatehi, Rhonda Drewes, Abby L. Grier, Monika Dzieciatkowska, Angelo D’Alessandro, Yongho Bae, Sachin A. Gupte

**Affiliations:** 1 Department of Pharmacology, New York Medical College, Valhalla, New York, USA; 2 Department of Pathology and Anatomical Sciences, Jacobs School of Medicine and Biomedical Sciences, University at Buffalo, State University of New York, Buffalo, New York, USA; 3 Department of Biochemistry and Molecular Genetics, University of Colorado Anschutz Medical Campus, Aurora, Colorado, USA

**Keywords:** epigenetics, vascular biology, smooth muscle cell, myocardin, plasminogen activator inhibitors, metabolic reprogramming, DNA methylation

## Abstract

The 3D genome architecture is a higher-order organization of chromosomes within the nucleus that is critical to the control of epigenomic modifications. However, our knowledge regarding the role of 3D genomic organization in the regulation of vascular gene expression and function is lacking. In the present study, CRISPR-engineered rats modelled after two common polymorphisms (S188F and N126D) in human glucose-6-phosphate dehydrogenase (G6PD) revealed modifications to the 3D genome in aortas from rats expressing a deficient G6PD variant (S188F), but not a non-deficient one (N126D), is associated with: 1] up-regulated expression of TET enzymes that augmented expression of genes encoding antiproliferative proteins, 2] suppressed expression of genes encoding inflammatory/thrombotic/fibrotic proteins, and 3] reduced angiotensin II-induced aortic stiffness and hypertension. G6PD interacted with MATRIN-3, a nuclear matrix/scaffold protein, and a deficient G6PD variant increased the relative abundance of MATR3 and CCCTC-binding factors, potentially modifying 3D-genome structure. Additionally, G6PD deficiency-induced enrichment of H3K27ac likely influences the establishment and maintenance of the 3D genome. Therefore, we propose that the nexus between metabolism and the 3D genome regulates arterial gene expression and vascular disease.

Epigenetic modification, a regulator of gene expression, is emerging as a switch that promotes the pathogenesis of diseases in humans, including vascular disease. DNA methylation and histone methylation or acetylation are epigenetic modifications now known to play critical roles in the pathogenesis of cancer and the development of vasculopathies, including hypertension. DNA methylation is regulated by DNA methyltransferases (DNMT: 1, 3a, and 3b) and DNA demethylases (methylcytosine dioxygenases TET: 1, 2, and 3) (1, 2). These enzymes are confined to the 3D space within the nucleus, where they are responsible for genetic imprinting and for the *de novo* regulation of DNA methylation, which regulates gene expression and function. Notably, emerging evidence suggests the spatial organization of the chromosomes within the eukaryotic nucleus may also contribute to the regulation of gene expression (3). In particular, DNA sequences and the 3D organization of the chromatin (chromosomal interactions) are considered to be important factors underlying gene function, in part by enabling interactions between distant fragments to regulate transactivation activity *via* mediator proteins (4). For instance, mapping genome-wide chromatin interactions using chromatin conformation capture technologies has demonstrated the importance of interacting domains such as topologically associated domains (TADs) and chromatin loops to the transcriptional regulation of genes in cancer cells (5, 6). Many such domains undergo changes during disease development and exhibit cell- and condition-specific differences (7). Modifications of 3D genomic organization can regulate gene expression. However, the mechanisms and protein mediators that regulate 3D genomic organization in vascular tissues are still unclear. Moreover, our knowledge regarding the 3D genomic basis of vascular function and vasculopathies is lacking. Therefore, one goal of this study was to determine whether interplay between 3D genome organization and DNA methylation regulates gene and vascular cell function. Another goal was to identify the protein that regulates 3D-genomic organization in vascular tissue.

Metabolism is critical to sustain life and metabolic reprogramming (Warburg phenomenon) is now emerging as a cause of many human ailments. Glucose-6-phosphate dehydrogenase (G6PD) is the rate-limiting enzyme of the pentose phosphate pathway, the main source of the reducing equivalent NADPH, a key cofactor that regulates antioxidant homeostasis through recycling of oxidized glutathione (8). However, the recent finding that G6PD is able to relocate to the nucleus of cells has implicated it in the regulation of gene function (9, 10). Interestingly, individuals with the Mediterranean G6PD polymorphism (S188F; G6PD^S188F^), a deficient variant with no change in protein expression but with only 10 to 20% residual activity (11), are less susceptible to cardio- and cerebrovascular diseases (12). By contrast, individuals with the African G6PD polymorphism (N126D; G6PD^N126D^), a non-deficient variant with no change in protein expression and 80% residual activity (11), are more susceptible to vascular disease and heart failure (13). Within the G6PD^S188F^ structure, an uncharged serine is substituted with a bulky hydrophobic phenylalanine, while in G6PD^N126D^ an uncharged asparagine is substituted with a negatively charged aspartic acid. We predict these substitutions alter the interaction between G6PD^S188F^ and G6PD^N126D^ and the chromatin. Furthermore, because S188 lies at the dimer-dimer interface of the solved canonical G6PD tetrameric structure (14), the two mutations may also impact the interaction between the chromatin and the G6PD multimer. This modified G6PD-chromatin interaction potentially alters the 3D genomic orientation, leading to the differential expression of genes encoding proteins that either reduces or augments the susceptibility of individuals to large artery (aortic) stiffness, thereby affecting Windkessel function and blood pressure.

Using CRISPR editing to model human G6PD^S188F^ and G6PD^N126D^ polymorphisms in rats, we demonstrate here that the deficient G6PD^S188F^ variant but not the nondeficient G6PD^N126D^ variant differentially modifies 3D-genome organization and upregulates expression of TET enzymes, which augments expression of genes encoding anti-proliferative/inflammatory proteins, suppresses expression of *Ccl5* and *Serpine1*, two genes encoding pro-inflammatory and pro-thrombotic/fibrotic proteins (CCL5 and PAI-1, respectively), decreases aortic stiffness and blood pressure, and reduces susceptibility to angiotensin II-induced aortic stiffness and hypertension. In addition, we show that G6PD^S188F^ rats exhibit TET-dependent hypomethylation and activation of Krüppel-like factor 13 gene (*Klf13*), encoding KLF13, which suppresses TGFβ-targeted activation of *Serpine1*, in the aorta. Finally, we show that application of *Serpine1* to the aorta *ex vivo* increases the elastic modulus, establishing PAI-1 as a mediator of arterial stiffness. Collectively, these results suggest that nuclear G6PD is a novel modifier of 3D genome organization that elicits TET expression and TET-dependent DNA demethylation, thereby regulating gene and cell function.

## Results

### Arterial metabolism is altered by G6PD polymorphism

**Figure 1:**
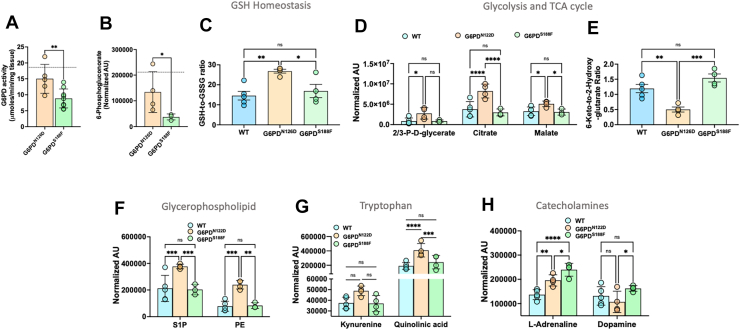
**Metabolism is altered in aortas from G6PD^S188F^ and G6PD^N126D^ rats.***A and B*, G6PD activity and 6-phosphogluconorate levels decreased more in aortas from G6PD^S188F^ than G6PD^N126D^ rats. The dashed line indicates G6PD activity and 6-phosphogluconorate levels in aortas from wild-type (WT) rats. *C-E*, Reduced glutathione (GSH) to oxidized glutathione (GSSG) ratio and intermediate metabolites in the glycolytic pathway and tricarboxylic acid cycle (TCA) were higher, and 6-ketoglutarate (6-Keto)-to-2-hydroxyglutarate (2-Hydroxy) ratio was lower in aortas from G6PD^N126D^ than G6PD^S188F^ or WT rats. *F, G*, intermediate metabolites in the glycerophospholipid biosynthesis, and indole and tryptophan pathway were higher in aortas from G6PD^N126D^ than G6PD^S188F^ or WT rats. *H*, metabolites in the catecholamine pathway were higher in aortas from G6PD^S188F^ and G6PD^N126D^ rats than WT rats (n = 5/group). Not significant (ns), ∗*p* < 0.05; ∗∗*p* < 0.005; ∗∗∗*p* < 0.001; and ∗∗∗∗*p* < 0.0005.

G6PD is a major enzyme in the pentose phosphate pathway branching from glycolysis and has been conserved throughout evolution (15). It irreversibly converts glucose-6-phosphate to 6-phosphogluconolactone within the pentose shunt, with concomitant generation of NADPH, which is in turn essential for redox homeostasis and anabolic reactions, such as fatty acid synthesis and other metabolic reactions relevant to cellular homeostasis (8, 15). We therefore first performed unbiased metabolomic analysis in G6PD^S188F^, G6PD^N126D^ and WT rats. As expected, G6PD activity and 6-phosphogluconorate levels were significantly lower in aortas from G6PD^S188F^ rats than from G6PD^N126D^ or wild-type [WT] rats, while no significant difference was observed between G6PD^N126D^ and WT rats (Fig. 1, *A* and *B*). Unexpectedly, the metabolomic results showed that the GSH-to-GSSG ratio (indicator of reductive stress) was higher (Fig. 1*C*), and the metabolites of the Krebs cycle, glycerophospholipid pathway and tryptophan pathway were up-regulated in aortas from G6PD^N126D^ rats as compared to G6PD^S188F^ and WT rats (Fig. 1, *D*–*G*). Interestingly, 6-ketoglutarate-to-2-hydroxyglutarate ratio was 2.4-fold lower in aortas from G6PD^N126D^ rats than WT rats and was 3-times higher in aortas from G6PD^S188F^ than G6PD^N126D^ rats (Fig. 1*E*). While L-adrenaline was higher in both G6PD^N126D^ and G6PD^S188F^ rats than WT rats, dopamine was higher in G6PD^S188F^ than G6PD^N126D^ rats (Fig. 1*H*). These findings indicate that the deficient G6PD^S188F^ and non-deficient G6PD^N126D^ polymorphisms differentially modify cellular metabolism.

### G6PD^S188F^ and G6PD^N126D^ variants differentially regulate gene expression

**Figure 2:**
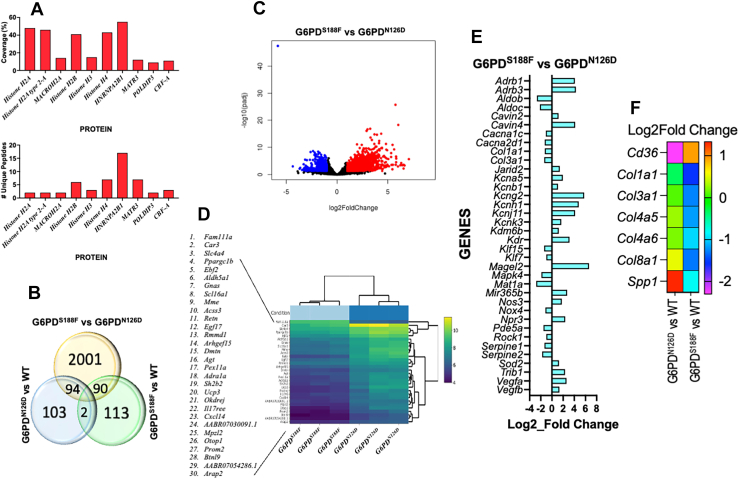
**Transcriptomic profiles are altered in aortas from G6PD^S188F^ and G6PD^N126D^ rats.***A*, RIME results showing G6PD interacts with proteins including histone. Coverage (*Top Panel*) and % unique peptides (*Bottom Panel*) identified by MS-TOF-TOF are shown. *B*, Venn diagram of unbiased RNAseq results showing differential gene expression among the three genotypes. *C-D*, Volcano plot and heat map showing differential up (*red*) and down (*blue*) regulation of genes in G6PD^S188F^*versus* G6PD^N126D^ rats. *E*, Expression of genes encoding proteins relevant to vascular structure and function, including ion channels, signaling proteins, lysine demethylases and transcription factors, in aortas from G6PD^S188F^ rats *versus* G6PD^N126D^ rats. *F,* Heat map showing that expression of genes encoding extracellular matrix collagen and osteopontin proteins and CD36, a membrane glycoprotein involved in fibrosis and inflammation was lower in aortas from G6PD^S188F^ rats [b] but not G6PD^N126D^ rats [a] than WT rats. N = 6/group.

G6PD is also the key rate-limiting enzyme that converts 6-carbon glucose to 5-carbon ribose and is involved in the biosynthesis of nucleoside substrates that fuel the *de novo* synthesis of RNA and DNA within cells (15). While G6PD is present predominantly in the cytoplasm, we and others have shown that a small amount of G6PD activity is present in the nucleus (9, 10, 16). In the present study, RIME analysis revealed that G6PD is associated with histones and other proteins, including nuclear matrix/scaffold proteins, within the nucleus (Fig. 2*A*). We therefore predicted that G6PD variants can modify cellular metabolism by regulating the activity or the expression of enzymes in the relevant metabolic pathways. To determine whether the G6PD variants modified metabolism by affecting enzyme expression, we performed unbiased transcriptomic analysis in aortas from the WT, G6PD^S188F^ and G6PD^N126D^ rats. Interestingly, although expression of 103 and 113 genes was differentially up- or downregulated in aortas from G6PD^N126D^ *versus* WT and G6PD^S188F^ *versus* WT rats, respectively, expression of 2001 genes was differentially up- or down-regulated in aortas from G6PD^S188F^ *versus* G6PD^N126D^ rats (Figs. 2*B* and S1). The volcano plot in Figure 2*C* shows that more genes were upregulated than down-regulated in aortas from G6PD^S188F^ than G6PD^N126D^ rats, and the heat map in Figure 2*D* shows the top up- and downregulated genes. Gene ontology of the top up- and downregulated genes indicate that these genes belonged to metabolic pathways, cell surface receptor and Rho signaling pathways, cell migration and growth pathways, and regulation of cell shape (Fig. S2). Furthermore, expression of genes related to vascular cell signaling and function (ion channels, cAMP signaling, MAPK/JNK signaling, endothelial growth, antioxidants, blood clotting, nitric oxide pathway) were also differentially up- or down-regulated (Fig. 2*E*). Moreover, the data revealed that genes encoding various subtypes of collagen (*Col1*, *Col3*, *Col4*, and *Col8*) and osteopontin (*Spp1*), proteins involved in the pathology of arterial stiffening, were significantly downregulated in G6PD^S188F^ rats as compared to G6PD^N126D^ *versus* WT rats (Fig. 2*F*). This suggests G6PD^S188F^ and G6PD^N126D^ differentially regulate genes encoding protein expression that potentially contribute to altering metabolism as well as the stiffness of the arteries.

### Expression of genes encoding pro-thrombotic/inflammatory/proliferative proteins is increased in aortas from G6PD^N126D^ rats, while expression of anti-proliferative and SMC-restricted proteins is increased in aortas from G6PD^S188F^ rats

**Figure 3:**
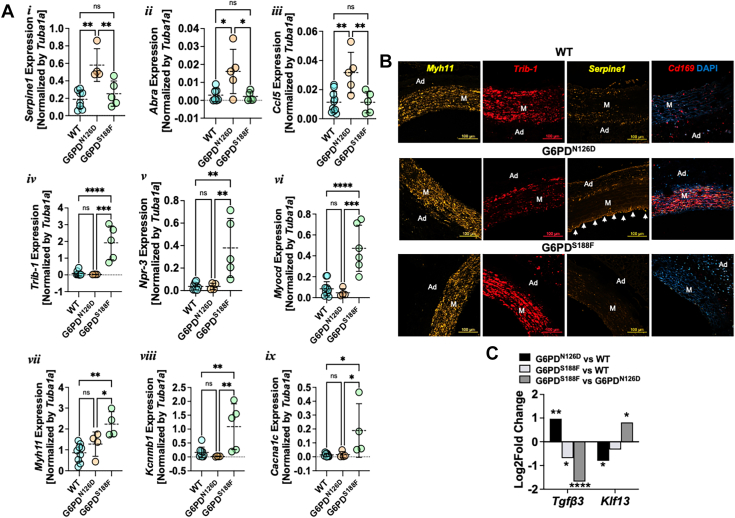
**Expression of genes encoding plasminogen activator inhibitor-1 and rantes proteins was increased in aortas from G6PD^N126D^ rats.***A*, expression of *Serpine1* (encoding PAI-1), *Ccl5* (encoding rantes), and *Abr*a (encoding a Rho activation protein) was higher in aortas from G6PD^N126D^ than WT rats. Expression of genes encoding smooth muscle cell-restricted proteins (*Myocd*, *Myh11*, *Kcnmb1* and *Cacna1c*), tribble-1 (*Trib1*) and natriuretic peptide receptor-3 (*Npr3*) was higher in aortas from G6PD^S188F^ than WT rats. *B*, representative images of *in situ* hybridizations showing expression of *Myh11*, *Trib1*, *Serpine1* and *Cd169* in the aortic wall (Ad: tunica adventitia and M: tunica media). *Trib1* and *Cd169* are expressed in the media, while *Serpine1* is predominantly expressed in intima and adventitia (Left 2 panels: *Myh11*, yellow; *Trib1*, red. Right 2 panels: Dapi, blue; *Serpine1*, yellow; *Cd169*, red) (n = 5/group). *C*, RNAseq results showing expression of *Tgfβ3* is higher, and *Klf13* is lower in aortas from G6PD^N126D^ than WT rats. Conversely, *Tgfβ3* expression is lower, and *Klf13* expression is higher in aortas from G6PD^S188F^ than G6PD^N126D^ rats. Not significant (ns), ∗*p* < 0.05; ∗∗*p* < 0.005; ∗∗∗*p* < 0.001; and ∗∗∗∗*p* < 0.0005.

To confirm the transcriptome results and differential expression of genes in aortas across the three genotypes, we performed qPCR with aorta samples from the same rats. The data confirmed greater expression of *Serpine1* in G6PD^N126D^ than G6PD^S188F^ or WT rats (Fig. 3Ai), which is consistent with the transcriptome results (Fig. 2*E*). In addition, expression of *Abra*, which encodes a Rho signaling protein that stimulates the serum response factor transcription pathway and promotes muscle cell proliferation and growth (17), and *Ccl5*, which encodes a proinflammatory protein (18), was higher in aortas from G6PD^N126D^ than G6PD^S188F^ or WT rats (Fig. 3Aii and Aiii). By contrast, expression of genes encoding signaling proteins (TRIB1; Fig. 3Aiv and NPR3; Fig. 3Av) and SMC-restricted proteins (MYOCD, MYH11, KCNMB1, CACNA1C; Fig. 3Avi to Aix) was higher in aortas from G6PD^S188F^ than G6PD^N126D^ or WT rats. To determine the location of *Serpine1* and *Trib1* expression in the aorta, we performed *in situ* hybridization using RNAscope. *Myh11* and *Trib1* were expressed in the media of aortas in all three genotypes (Fig. 3*B*). However, although *Serpine1* was expressed in the aortic wall in all three genotypes, its expression was weaker in aortas from G6PD^S188F^ than WT or G6PD^N126D^ rats. Moreover, bright *Serpine1* staining was also observed at the media-intima and media-adventitia borders in aortas from G6PD^N126D^ rats (Fig. 3*B*). On the other hand, *Ccl5* expression was detected in the intima and adventitia (Fig. S3). In addition, to determine the potential source of inflammatory cytokines/chemokines, we performed *in situ* hybridization for *Cd169*, which encodes CD169, a marker of tissue-resident macrophages (19, 20), and detected stronger *Cd169* signals in the media of aortas from G6PD^N126D^ rats than WT or G6PD^S188F^ rats. Indeed, there was no detectable *Cd169* signal in aortas from G6PD^S188F^ rats. This indicates that expression of genes encoding pro-thrombotic/inflammatory proteins and resident macrophage markers is increased in the media and intima of aortas from G6PD^N126D^ rats. To then determine a potential cause of the higher *Serpine1* and cytokine expression in aortas from G6PD^N126D^ than WT or G6PD^S188F^ rats, we re-examined our transcriptome results and found that the expression of the gene encoding KLF13, which is a negative regulator of TGFβ-targeted genes, including *Col1a* and *Serpine1* (21), was weaker in aortas from G6PD^N126D^ than WT rats and stronger in aortas from G6PD^S188F^ than G6PD^N126D^ rats (Fig. 3*C*). By contrast, expression of the gene encoding TGFβ3, one of the three isoforms that play a critical role in the synthesis and degradation of extracellular matrix (ECM) (22), was higher in aortas from G6PD^N126D^ than WT rats and lower in aortas from G6PD^S188F^ than G6PD^N126D^ rats (Fig. 3*C*).

### *Klf13* was hypomethylated while *Tgfβ3* and *Serpine1* were hypermethylated in aortas from G6PD^S188F^ but not G6PD^N126D^ rats

**Figure 4:**
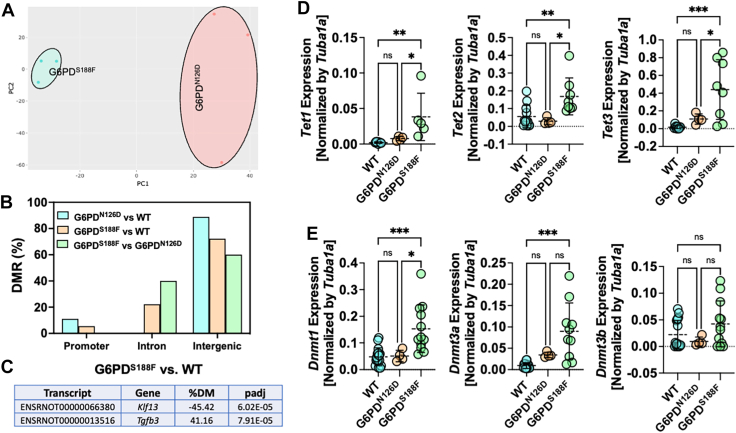
**DNA is differentially methylated, *Klf13* promoter is hypomethylated, and expression of genes encoding DNA demethylases is increased in aortas from G6PD^S188F^ but not G6PD^N126D^ rats.***A*, Principal component plot showing that DNA is differentially methylated between aortas from G6PD^S188F^ and G6PD^N126D^ rats. *B*, Whole-genome bisulfite sequencing showing that CpG island methylation in the promoter and intergenic differs less between aortas from G6PD^S188F^ and WT rats and between G6PD^S188F^ G6PD^N126D^ rats than between G6PD^N126D^ and WT rats. *C*, the *Klf13* promoter is hypomethylated and *Tgfβ3* promoter is hypermethylated in aortas from G6PD^S188F^ as compared to WT rats. N = 5/group in *A*-*C*. *D*, Expression of genes encoding ten-eleven translocation (Tet) methylcytosine dioxygenases 1(*Tet1*, *Tet2*, and *Tet3*) is higher in aortas from G6PD^S188F^ than G6PD^N126D^ or WT rats. *E*, expression of genes encoding DNA methyltransferase 1 and 3a (*Dnmt1* and *Dnmt3a*) but not 3b (*Dnmt3b*) is higher in aortas from G6PD^S188F^ than WT rats (n = 5–10/group). Not significant (ns), ∗*p* < 0.05; ∗∗*p* < 0.005; and ∗∗∗*p* < 0.001.

It is well established that gene expression is both transcriptionally and post-transcriptionally regulated. DNA methylation-demethylation is an off-on switch that governs gene transcription (23). To determine whether DNA methylation contributes to the difference in the gene expression profiles between G6PD^S188F^ and G6PD^N126D^ variant rats, we performed whole-genome bisulfite-sequencing with aortas from WT, G6PD^N126D^, and G6PD^S188F^ rats. The PCA plot in Figure 4*A* shows differences in DNA methylation between aortas from G6PD^S188F^ and G6PD^N126D^ rats. We also detected differences in aortic DNA methylation between G6PD^S188F^ and WT rats but not between G6PD^N126D^ and WT rats (Fig. S4). Quantification of the methylation of CpG islands and loci across the whole genome indicated less methylation of CpG islands in promoter and intergenic regions in G6PD^S188F^ than WT or G6PD^N126D^ rats but no significant difference between G6PD^N126D^ and WT rats (Fig. 4*B*). Moreover, we found that *Klf13* was hypomethylated and *Tgfβ3* was hypermethylated in aortas from G6PD^S188F^ as compared to WT rats (Fig. 4*C*), while no such differences were observed between G6PD^N126D^ and WT rats. Likewise, the *Serpine1* promoter was also hypermethylated in G6PD^S188F^ rats (Fig. S5). Thus, the G6PD^S188F^ and G6PD^N126D^ variants are associated with different DNA methylation and gene expression profiles in vascular tissue.

### Expression of *Tets* is increased in the aortas from G6PD^S188F^ but not G6PD^N126D^ rats

**Figure 5:**
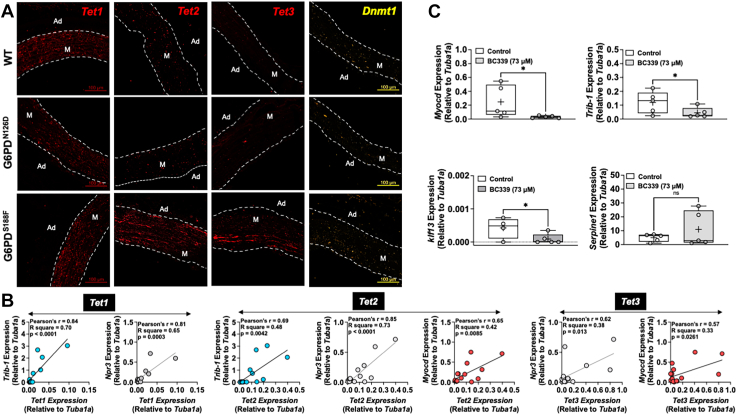
***Tet2* expressed in the aortic media increases expression of *Myocd* and *Trib1* and decreases arterial stiffness in rats.***A*, representative image of *in situ* hybridization showing *Tet1*, *Tet2*, *Tet3* and *Dnmt1* expressing in the aortic media (M: tunica media and Ad: tunica adventitia) in WT, G6PD^N126D^ and G6PD^S188F^ rats. While *Tet1* and *Dnmt1* are expressed in the media of all three genotypes, expression of *Tet2* and *Tet3* is higher in G6PD^S188F^ than G6PD^N126D^ or WT rats. (*Tet1*, *Tet2*, and *Tet3* is shown in *red*; *Dnmt1* is in *yellow*) (n = 5/group). *B*, correlation analysis showing positive Pearson’s correlation between expression of *Trib1*/*Npr3*/*Myocd versus Tet1*/*Tet2*/*Tet3*. *C*, application of BC339 (73 μM), a TET activity inhibitor, to aortas isolated from WT and G6PD^S188F^ rats and cultured *ex vivo* for 72 h reduced expression of *Myocd, Trib1* and *Klf13* but not *Serpine1*. Not significant (ns), ∗*p* < 0.05; ∗∗*p* < 0.005; ∗∗∗*p* < 0.001; and ∗∗∗∗*p* < 0.0005.

To determine why methylation of CpG regions differed between the genotypes, we quantified the expression of genes encoding TETs and DNMTs. Notably, expression of all three *Tets* (*1*, *2* and *3*) as well as *Dnmt1* and *Dnmt3a*, but not *Dnmt3b*, was higher in aortas from G6PD^S188F^ than G6PD^N126D^ or WT rats (Fig. 4, *D* and *E*). Additionally, *in situ* hybridization (RNAscope) revealed the presence of *Tet* (*1*, *2* and *3*) and *Dnmt1* mRNA in the media of aortas from G6PD^S188F^ rats (Fig. 5*A*). This suggests increased expression of demethylases led to hypomethylation of CpG islands in the genome of G6PD^S188F^ rats as compared to G6PD^N126D^ and WT rats.

### Expression of *Myocd* and *Trib1* is augmented in a TET-dependent manner in aortas from G6PD^S188F^ rats

Because expression of *Tet* genes and the extent of DNA hypomethylation were greater in G6PD^S188F^ than G6PD^N126D^ rats, we performed Pearson’s R correlations to determine the relationship between the expression of individual *Tets* and *Myocd*/*Trib1*/*Npr3*. We found a positive correlation (*p* < 0.05) between 1] *Trib1*/*Npr3* and *Tet1*; 2] *Trib1*/*Npr3*/*Myocd* and *Tet2*; and 3] *Npr3*/*Myocd* and *Tet3* (Fig. 5*B*). Application of a TET inhibitor, 1-([1,1′-biphenyl]-3-yl)-4-amino-5-chloropyrimidin-2(1H)-one (BC339; 73 μM), which inhibits TET2 activity (24), to aortas from WT rats cultured *ex vivo* for 72 h decreased expression of *Myocd*, *Trib1*, and *Klf13* but not *Serpine1* (Fig. 5*C*). These results indicate that TET2-dependent hypomethylation contributes to increased expression of genes encoding *Myocd*, *Trib1*, and *Klf13* in aortas from G6PD^S188F^ rats.

### 3D genome organization, including topologically associated domains and chromatin loops in *Tet* genes, is modified by G6PD^S188F^ polymorphism

**Figure 6:**
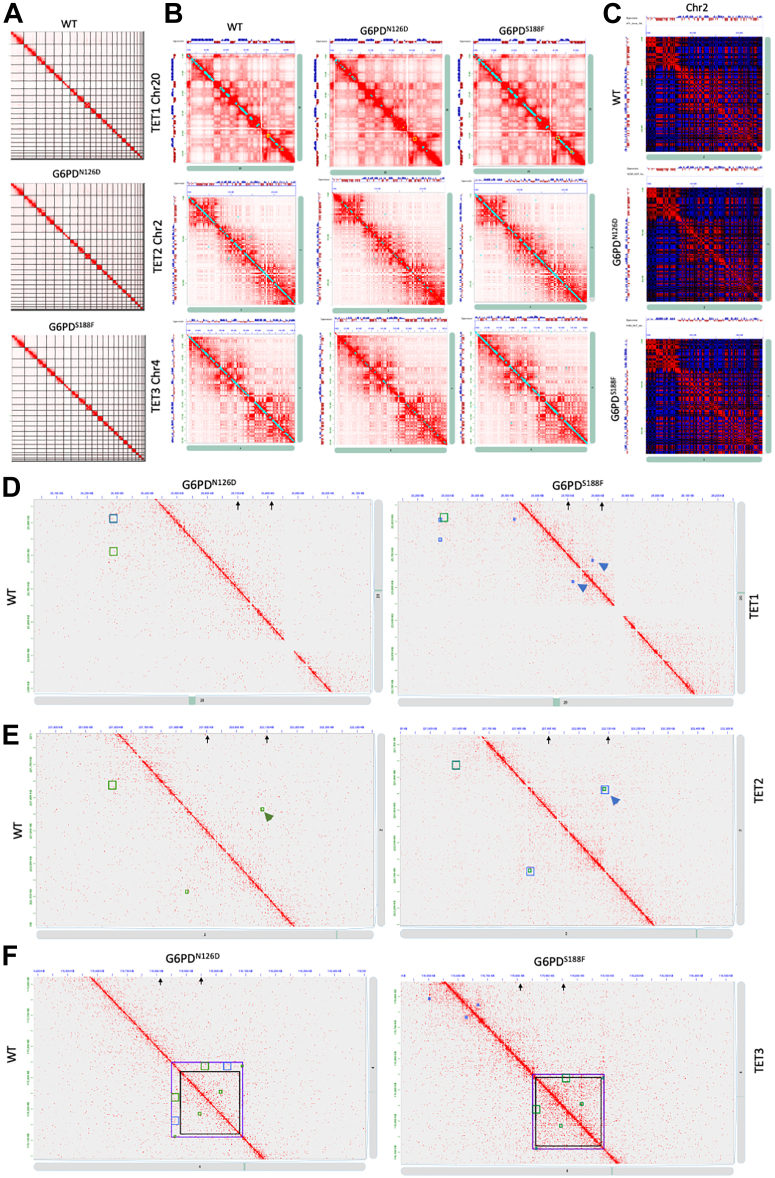
**Whole Genome HiC heatmaps of WT, G6PD^S188F^ and G6PD^N126D^ rat aortas.***A*, representative HiC images generated for chromosomes 1 through 20 and chromosomes X and Y (n = 3/group). The x-axis and y-axis are genomic coordinates and each pixel in the heatmap represents the number of proximally ligated molecules sequenced between those two places in the genome. This is a measure for how frequently any given two loci interact with each other in 3D space. The intensity of the red corresponds to the HiC counts sequenced between the two loci and the frequency of said loci in the nucleus. Red is higher counts while white is zero counts. Each off-diagonal square indicates one chromosome’s interactions with another chromosome (Trans), with the diagonal squares representing a single chromosome interacting with itself (Cis). *B*, images zoomed in to chromosome 20, 2 and 4 from ∼26 Mb – 52 Mb. Chromatin loops and topologically associating domains (TAD) are overlaid with loop calls (cyan squares) and TAD calls (yellow squares) from Juicebox (Juicer HiCCUPS). The A/B compartment signal from Juicer Eigenvector plotted in the wiggle format in blue (positive values represent compartment A or euchromatin) and red (negative values represent compartment B or heterochromatin) are the *left* and *top* of the figure. *C*, Pearson’s correlation plot of chromosome 2 depicts linear positive (*blue*) and negative (*red*) interactions. *D-F*, representative .hic files zoomed in to 1 KB resolution for chromosomes 20 (*D*; *Tet1*), 2 (*E*; *Tet2*) and 4 (*F*; *Tet3*). 2D annotation analysis shows chromatin loops (*small blue squares*) and TADs (*large black squares*) for G6PD^S188F^ and G6PD^N126D^ rats, and chromatin loops (*small green squares*) and TADs (*large purple squares*) for the control WT. The color range is 0 to 14 for all images. Merged comparisons include WT and G6PD^S188F^ and WT and G6PD^N126D^. It is noteworthy that two loops (*green* and *blue squares*) are present in the *Tet1* and *Tet2* regions in G6PD^S188F^ (*blue arrowhead*) *versus* WT but not G6PD^N126D^ (*green arrowhead*) *versus* WT. While TADs were present in both G6PD^S188F^ and G6PD^N126D^ rats, the number and position of the loops in the *Tet3* region appear to differ between the two genotypes in comparison to WT (*Bottom panels*).

**Figure 7:**
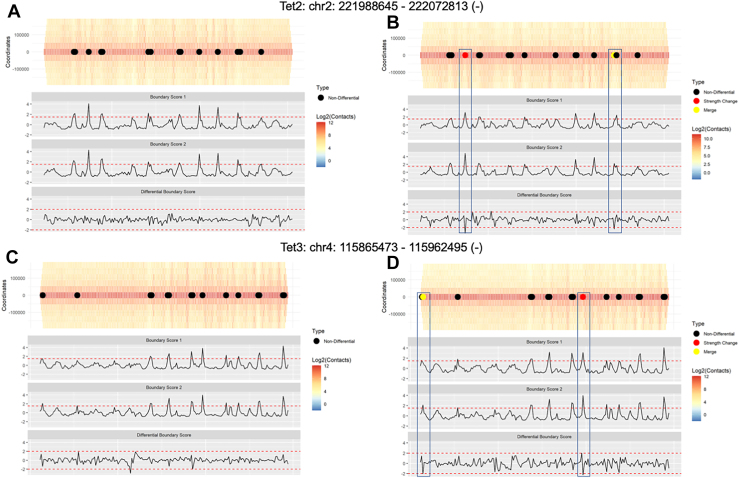
**Temporal analysis of TADs and differential analysis of boundaries of interacting domains between HiC datasets.** (*A, C)* WT *versus* G6PD^S188F^ and (*B*, *D*) WT *versus* G6PD^N126D^ comparison of TAD and the TAD boundaries of interacting domains are shown using TADcompare R package. *Black solid* circles indicate non-differential boundaries, red circles indicate strength change boundaries, and yellow circles indicate merged boundaries in the chromosome 2 segments where *Tet2* and *Tet3* are located.

DNA and chromosomes are compacted and packaged within the eukaryotic nucleus. Consequently, the spatial organization of chromosomes is important for DNA replication, repair, and gene expression (3). A recent study suggests DNA methylation modifies the 3D genomic organization (25). HiC sequencing, the state-of-the-art approach to producing a 3D map of chromosomal organization that enables genome-wide unbiased capture of chromatin interactions (5), revealed that within the genome, there are differences in chromatin loops and TADs between G6PD^S188F^ and WT rats and between G6PD^N126D^ and WT rats (Fig. 6, *A*–*C*). The eigenvector results shown in Figure 6, *B* and *C* indicate that compartments A (positive values in blue on top and side of chromosome) and B (negative values in red on top and side of chromosome), which correspond to the open (euchromatin) and closed (heterochromatin) chromatin structures, are present in chromosomes 20, 2 and 4, respectively harboring *Tet1*, *Tet2* and *Tet3*, in all three genotypes. However, a B-to-A compartment change was seen in chromosome 2, including the region near *Tet2*, in G6PD^S188F^ rats as compared to G6PD^N126D^ or WT rats (Fig. 6, *B* and *C*). Pearson’s correlation showed linear positive (red) and negative (blue) interactions in chromosome 2 in all three genotypes (Fig. 6*C*). Further, high-resolution 3D annotation analysis showed formation of new chromatin loops (small blue squares) within *Tet1* coordinates on chromosome 20 of G6PD^S188F^ but not G6PD^N126D^ rats when compared to WT rats (Fig. 6*D*). Similarly, new chromatin loops (small blue squares) were detected within the coordinates of *Tet2* on chromosome 2 in G6PD^S188F^ but not G6PD^N126D^ rats, in addition to chromatin loops (small green square) in the control WT rats (Fig. 6*E*). 3D annotation analysis also showed chromatin loops (small blue squares) and TADs (large black squares) in the G6PD^S188F^ and G6PD^N126D^ rats and chromatin loops (small green squares) and TADs (large purple squares) in control WT rats (Fig. 6*F*). We then analyzed the HiC dataset using a modified TADCompare program (Fig. 7), an R language package used for temporal analysis of TADs and differential analysis of the boundaries of interacting domains between two or more HiC datasets (7). That analysis revealed differences between the boundary scores of TADs in aortas from G6PD^N126D^ and WT rats (Fig. 7*B*) but not between G6PD^S188F^ and WT rats (Fig. 7*A*). Notably, in chromosome 2 segment 221,988,645 - 222,072,813 (−), where *Tet2* is located, we found non-differential boundaries in aortas from G6PD^S188F^ *versus* WT rats (Fig. 7*A*). By contrast, we found merged and strength change boundaries in addition to non-differential boundaries in aortas from G6PD^N126D^ *versus* WT rats (Fig. 7*B*). Similarly, in chromosome 4 segment 115,865,473 to 115,962,495 (−), where *Tet3* is located, we found merged and strength change boundaries in addition to non-differential boundaries in aortas from G6PD^N126D^ *versus* WT rats (Fig. 7*D*) but only non-differential boundary changes in G6PD^S188F^ *versus* WT rats (Fig. 7*C*).

In addition, HiC results also revealed new chromatin loop and/or TAD boundary formation within the genome of G6PD^S188F^ but not G6PD^N126D^ rats as compared to WT rats around the *Nos3* and *Cacna1c* segments on chromosome 4 (Fig. S6), *Trib1* segments on chromosome 7 (Fig. S7), *Myocd* segments on chromosome 10 (Fig. S8) and *Lmod1* on chromosome 13 (Fig. S9). While new chromatin loop formation was observed within the genomes of G6PD^S188F^ and G6PD^N126D^ rats around *Serpine1* on chromosome 12, no new loops were found near *Ccl5* on chromosome 10 (Fig. S10). Additionally, compared to WT rats, we found new chromatin loops around *G6pd* on chromosome X in G6PD^S188F^ but not G6PD^N126D^ rats (Fig. S11). Overall, these results indicate that modifications in the 3D genomic organization and new chromatin loop formation evoked by the G6PD^S188F^ variant, activates TET2 expression, which increases transcription of genes encoding vasculo-protective proteins (MYOCD, TRIB1, and KLF13) involved in preventing SMC dedifferentiation and reducing the vascular damage.

### Relative abundance of MATRIN-3 (MATR3) and CCCTC-binding factor (CTCF) is increased by G6PD^S188F^ polymorphism

**Figure 8:**
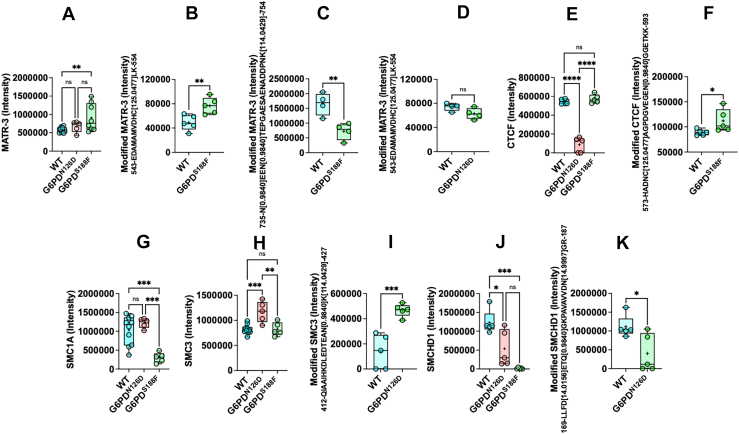
**Expression of MATRIN-3 (MATR3) and CCCTC-binding factor (CTCF) increased in G6PD^S188F^ rats.***A*, quantitative Mass-Spectrometry-based proteomic analysis revealed a higher expression of MATR3 in G6PD^S188F^ rats than in G6PD^N126D^ and wild-type (WT) rats. *B, C*, relative abundance of MATR3 with reduced Cys (125.0477) residues is more and with di-Gly modification on lysine (K[114.0429]) residue is less in G6PD^S188F^ rats as compared with WT rats. *D*, expression of MATR3 with reduced Cys (125.0477) residues is not different in G6PD^-N126D^ rats from WT rats. *E*, CTCF is expressed less in G6PD^N126D^ rats than in WT and G6PD^S188F^ rats. *F*, relative abundance of CTCF with reduced Cys (125.0477) and deamidation of Asn (N[0.9840]) residue is more in G6PD^S188F^ rats compared with WT rats. *G, H*, expression levels of SMC1A and SMC3, cohesion subunits, are lower in G6PD^S188F^ than in G6PD^N126D^ rats. (*I*) Relative levels of SMC3 with deamidation of Asn (N[0.9840]) and di-Gly lysine (K[114.0429]) residue is more in G6PD^N126D^ rats compared with WT rats. *J, K*, expression of SMCHD1 is decreased in G6PD^N126D^ and G6PD^S188F^ rats than in WT rats, as well as the relative abundance of SMCHD1 with modified Asp (D[14.0156]), deamidation Gln (Q[0.9840]), and Asn to Asp methylation (N[14.9997]), residues are reduced in G6PD^N126D^ than in WT rats. N = 4 to 5/group, not significant (ns), ∗*p* < 0.05; and ∗∗*p* < 0.005.

Recently, two major modes of 3D folding of the genome and the mechanisms that drive its multilayered organization in mammalian genomes, namely CTCF/cohesin-dependent and chromatin state-driven mechanisms, have emerged (26). MATR3, a nuclear matrix/scaffold protein, interacts with CTCF and the cohesin complex that is crucial in organizing TADs and mediating chromatin interactions of individual genomic loci (27). Loss of MATR3 perturbs 3D-genome structure (27). By RIME analysis, we found that G6PD interacts with various histones and MATR3 (Fig. 2*A*). Additionally, by performing quantitative Mass-Spec based proteomics, as described previously (28, 29), we found higher relative abundance of MATR3 (Fig. 8, *A*–*D*) and CTCF (Fig. 8*E*) protein with reduced Cys residue (C[125.0477]; Fig. 8*F*), and lower relative abundance of MATR3 peptide with di-Gly modification on lysine residue (K[114.0429]), which indicates remnant from ubiquitination after trypsin digestion in Mass-Spec., in G6PD^S188F^ rats (Fig. 8*C*). Relative abundance of MATR3 with reduced Cys residues remained unchanged in G6PD^N126D^ rats (Fig. 8*D*).

### Differential expression and post-translational modifications of cohesin protein subunits between genotypes

Proteomic analysis revealed lower abundance of the cohesin subunits SMC1A and SMC3 in G6PD^S188F^ rats compared with G6PD^N126D^ rats (Fig. 8, *G* and *H*). While no significant post-translational modifications (PTMs) were detected in SMC1A, SMC3 exhibited significant PTMs in G6PD^N126D^, but not G6PD^S188F^, rats relative to WT controls (Fig. 8*I*). These modifications included increased Asn424 (N[0.984]), indicative of amino acid deamidation, and Lys425 (K[114.0492]), indicative of GlyGly remnant from ubiquitination. Intriguingly, we also observed that the relative abundance of SMCHD1—an SMC-like protein that anchors heterochromatin domains to the nuclear lamina and restricts their accessibility to DNA methyltransferases and other epigenetic modifiers typically associated with active chromatin, and which has been shown to influence genome architecture (30)—was decreased significantly more in G6PD^S188F^ rats than in G6PD^N126D^ rats compared with WT controls (Fig. 8*J*). In addition, G6PD^N126D^ rats exhibited reduced relative abundance of SMCHD1 carrying PTMs at Gln175 (N[0.984]) and Asn (N[14.997]), consistent with amino acid methylation, relative to WT rats (Fig. 8*K*).

### Knockdown of SMC3 inhibits Tet2 expression

**Figure 9:**
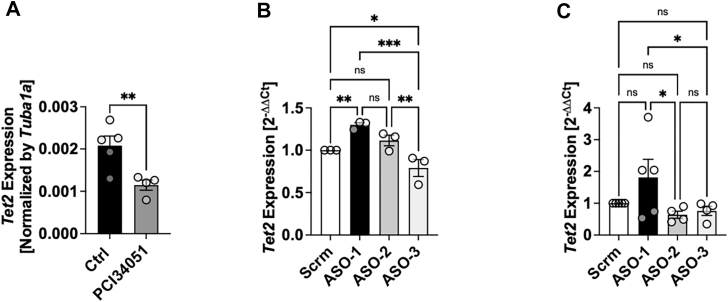
**Perturbation of cohesin function reduces *Tet2* expression.** (*A*) Inhibition of SMC3 deacetylation with the HDAC8-specific inhibitor PCI-34051 (10 nM; IC_50_) significantly reduced *Tet2* mRNA expression in cultured aortic smooth muscle cells (SMCs) isolated from wild-type (WT) rats. *B*, AUM*silence* sdASO–mediated knockdown of *Smc3* decreased *Tet2* expression in cultured WT rat SMCs. *C*, targeted knockdown of *Smc3* using sdASOs similarly reduced *Tet2* expression in aortas from G6PD^S188F^ rats. Data are presented as mean ± SEM. Statistical significance was determined using [Student’s *t* test or ANOVA, as appropriate]; *p* < 0.05 was considered significant. ∗*p* < 0.05; ∗∗*p* < 0.01; ∗∗∗*p* < 0.005.

Next, we assessed whether perturbation of chromatin configuration alters *Tet2* expression. Cohesin subunits, including SMC1A and SMC3, organize chromosomes by extruding DNA loops to form TADs (31). Because acetylation of SMC3 restricts chromatin loop length and the extension of architectural stripes emanating from CTCF sites – thereby regulating three-dimensional genome organization (32) – we inhibited SMC3 deacetylation using the HDAC8-specific inhibitor PCI-34051 (10 nM; IC_50_) (33). Treatment with PCI-34051 significantly decreased *Tet2* expression in cultured SMCs from WT rats (Fig. 9*A*). We next assessed the effect of direct SMC3 knockdown on *Tet2* expression. AUM*silence* sdASO–mediated targeted knockdown of SMC3 (5 uM) (Table 1) similarly reduced *Tet2* expression in cultured SMCs from WT rats (Fig. 9*B*) and in aortas from G6PD^S188F^ rats (Fig. 9*C*). Although the precise reason why ASO#2 and ASO#3 reduced *Tet2* expression more effectively in aorta and SMCs, respectively, than ASO#1 and ASO#4 (data not shown) is not fully understood, this effect likely reflects more efficient *Smc3* knockdown (Table 1) and/or improved cellular penetration of these ASOs.

**Table 1:** *Smc3*-trageting sdASOs reduce *Smc3* mRNA levels

*Smc3*-sdASO#	Sequence	*Smc3* mRNA levels (2^−ddCT^) mean ± SEM
1	TCTCTGTTGGGTCTCTAT	0.48 ± 0.13
2	TCCCGTTTGCCCTCTGTTTCT	0.16 ± 0.06
3	GCCATTTGGTTGATCTTTCC	0.16 ± 0.05
4	TCTCTATCTGCTGCATTTGG	0.36 ± 0.09

### Histone acetylation is increased by G6PD^S188F^ polymorphism

**Figure 10:**
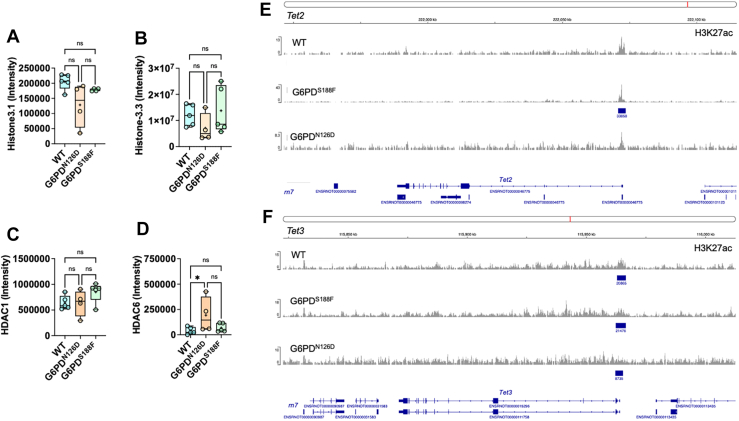
**Histone acetylation is increased by the G6PD^S188F^ variant.***A-D*, Mass-spectrometry-based proteomic analysis showing relative abundance of histone (3.1 and 3.3) and histone deacetylase 1 and 6 (HDAC1 and HDAC6) in all three genotypes. *E*, *F*, Cut&Run analysis revealed H3K27ac enrichment in promoter regions close (*blue block* under the peak) to the first exons of *Tet2* (*E*; top indicated by red line) and *Tet3* (*F*; *top indicated by red line*). Visualized using the IGV browser (n = 3/group). Not significant (ns) and ∗*p* < 0.05.

Histone acetylation is associated with loops and likely influences the establishment and maintenance of TADs and loops in the genome (5). We previously showed that G6PD directly interacts with HDAC5 (34) and that inhibition of G6PD activity or knockdown of G6PD protein in SMC decreases HDAC activity and increases acetylation of H3K9 and H3K27 (35). In the present study, we found G6PD interacts with histones (Fig. 2*A*). Moreover, loss of SMCHD1 increases H3K27ac mark on the loop and promotes the B-to-A transition indicating open chromatin (30). By proteomic analysis we determined whether G6PD deficiency affects the expression levels of histones and HDACs. Although there was no difference in the expression of histone (3.1 and 3.3) and HDAC1 between three genotypes (Fig. 10, *A*–*C*), we found a higher expression of HDAC6 in G6PD^N126D^ but not G6PD^S188F^ rats as compared with WT rats (Fig. 10*D*). Furthermore, reactive oxygen species directly or indirectly inactivates class I HDACs (36, 37). Therefore, since reactive oxygen species production is altered by the changes in NADPH and GSH redox, we determined whether G6PD variant-induced redox modifies histone acetylation in the genome by performing CUT&RUN. Our results indicated that there was increased H3K27ac enrichment on *Tet2* in G6PD^S188F^ rats (Fig. 10*E*). There was also H3K27ac enrichment on *Tet3* in all three genotypes, though the magnitude was greater in G6PD^S188F^ rats (Fig. 10*F*).

### Aortic stiffness and blood pressure are higher in G6PD^N126D^ than G6PD^S188F^ or WT rats

**Figure 11:**
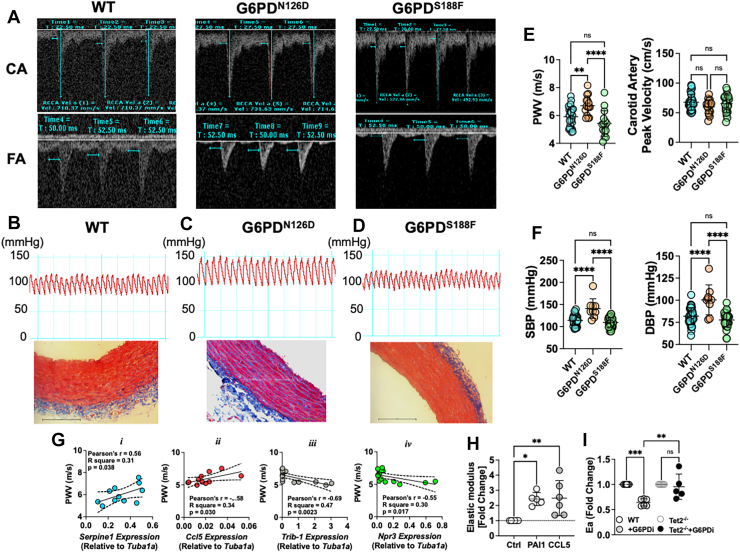
**Pulse wave velocity and blood pressure are decreased in G6PD^S188F^ but not G6PD^N126D^ rats.***A*, representative echocardiographs showing the blood flow in carotid (CA) and femoral (FA) arteries of WT, G6PD^N126D^ and G6PD^S188F^ rats. *B-D*, representative blood pressure traces (*top*) and images of aorta stained with Mason’s Trichrome (*bottom*) in WT, G6PD^N126D^ and G6PD^S188F^ rats. *E*, PWVs calculated as the distance (cm) between the carotid and femoral arteries - divided by time (s) were slower in G6PD^S188F^ than in G6PD^N126D^ or WT rats. The peak PWV in the common carotid artery did not differ among the three genotypes. *F*, systolic and diastolic blood pressures were lower in G6PD^S188F^ than in G6PD^N126D^ rats. N = 15 to 20/group. *G*, Pearson’s correlation between pulse wave velocity (PWV) and expression of *Serpine1*, *Ccl5*, *Trib1* and *Npr3*. Positive correlations between PWV *versus Serpine1*/*Ccl5* and negative correlations between PWV *versus Trib1*/*Npr3* were found. *H*, atomic force microscopy showed that application of PAI-1 (50 ng/ml) and CCL5 (1 mg/ml) to aortas *ex vivo* for 72 h increased the elastic modulus, an indicator of stiffness (n = 5–6/group). *I*, subcutaneous injection of N-ethyl-N-[(3,5)-17-oxoandrostan-3-yl]urea (PD2958; 1.5 mg/kg/day; sc), a G6PD inhibitor (42), to mice for 3 weeks, reduced arterial elastance (indicator of arterial stiffness) in WT (n = 7/group) but not Tet knockout (*Tet2*^*−/−*^; n = 5/group) mice. Not significant (ns), ∗*p* < 0.05; ∗∗*p* < 0.005; ∗∗∗*p* < 0.001; and ∗∗∗∗*p* < 0.0005.

G6PD deficiency is the most common enzymopathy in humans, affecting approximately 500 million people around the world (38). Epidemiological studies suggest that individuals from the Mediterranean region carrying the deficient G6PD^S188F^ polymorphism are less susceptible to cardiovascular diseases (12, 15) and cancer (15, 39), whereas individuals from the sub-Saharan African region and Africans in other parts of the world carrying the non-deficient G6PD^N126D^ polymorphism (11), are more susceptible to vascular diseases and heart failure (13). We therefore compared the vascular function of G6PD^S188F^ and G6PD^N126D^ rats to that of WT rats. Echocardiographic studies revealed that G6PD^N126D^ rats had a higher pulse wave velocity (PWV; a determinant of aortic stiffness that increases with stiffness and is directly proportional to stiffness) than WT or G6PD^S188F^ rats (Fig. 11, *A* and *E*). Although trichrome staining (blue) revealed the presence of collagen in the adventitia in all three genotypes, collagen deposits were also seen in the interstitial spaces in the media of aortas from G6PD^N126D^ rats (Fig. 11*C* bottom panel) but not WT (Fig. 11*B* bottom panel) or G6PD^S188F^ rats (Fig. 11*D* bottom panel). G6PD^N126D^ rats (Figs. 11*C* top panel and -11*F*) also had higher systolic and diastolic blood pressures than G6PD^S188F^ (Fig. 11*D* top panel and 11*F*) or WT (Fig. 11*B* top panel and 11*F*) rats. These results suggest that the arteries from rats carrying the G6PD^N1226D^ polymorphism are stiffer and less compliant than those from the other two genotypes, leading to higher blood pressures. It is therefore likely that G6PD^N1226D^ rats are more predisposed to develop vascular diseases than G6PD^S188F^ or WT rats.

### Upregulated *Serpine1* expression correlates positively with the increased pulse wave velocity and PAI-1 and CCL5 increases elastic modulus, and TET2 mediates G6PD inhibitor-induced decreases in aortic stiffness in mice

Having detected differentially modified -3D organization of the genome and differential expression of genes encoding proteins that potentially influence the phenotype and function of vascular cells in G6PD^S188F^ *versus* G6PD^N126D^ *versus* WT, we next assessed the correlation between gene expression and aortic stiffness. We found PWV correlated positively with expression of *Serpine1* and *Ccl5* (Fig. 11, *Gi*-*ii*) but negatively with expression of *Trib1* and *Npr3* (Fig. 11, *Giii*-*iv*). This indicated that *Serpine1* and *Ccl5* encode proteins that potentially contribute to the aortic stiffening seen in G6PD^N126D^ rats. We therefore tested whether PAI-1 and CCL5 increase aortic stiffness. We applied PAI-1 (50 ng/ml) and CCL5 (1 mg/ml) to aortas isolated from WT rats and cultured *ex vivo* for 3 days, and then used atomic force microscopy (AFM) to measure the elastic modulus. Interestingly, both PAI-1 and CCL5 significantly increased the elastic modulus over 2-fold as compared to vehicle controls (Fig. 11*H*). This suggests that increased expression of PAI-1 and CCL5 contributes to the increased aortic stiffness.

Next, because the TET inhibitor decreased expression of genes encoding SMC-restricted MYOCD, which inhibits SMC dedifferentiation (40), and the cell signaling protein TRIB1, which inhibits SMC proliferation (41), in aortas from WT rats, we wanted to test whether elevated TET2 prevented vascular stiffness in G6PD^S188F^ rats. Therefore, we determined whether inhibiting G6PD activity reduces arterial stiffness through TET2 signaling. Subcutaneous injection of N-ethyl-N-[(3,5)-17-oxoandrostan-3-yl]urea (NEOU; 1.5 mg/kg/day; sc), a G6PD inhibitor (42), to mice for 3 weeks decreased arterial elastance [Fig. 11*I*; an indicator of arterial stiffness determined by inserting catheter in left ventricle as described previously (43)] and blood pressure (Fig. S12) in WT but not in *Tet2*^*−/−*^ mice. These results indicate that TET2-dependent hypomethylation contributes to G6PD inhibition-induced decrease of arterial stiffness in mice.

### Ang II-induced aortic stiffness and hypertension are exacerbated in G6PD^N126D^ but not G6PD^S188F^ rats

**Figure 12:**
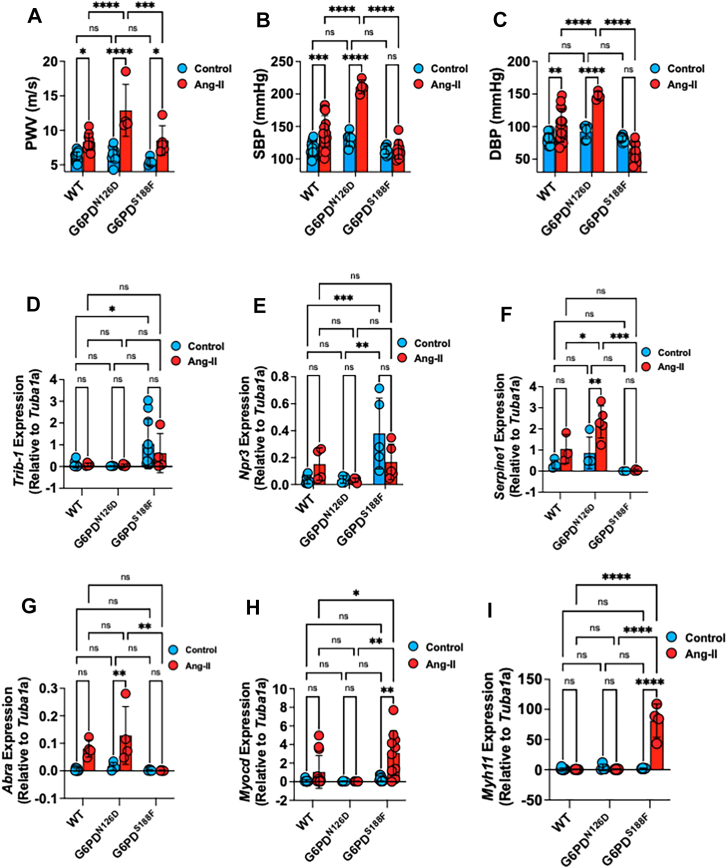
**Angiotensin II increases arterial stiffness and blood pressure in G6PD^N126D^ but not G6PD^S188F^ rats.***A-C*, Ang II infusion (400 ng/kg/day s.c.) induced greater increases in PWV and systolic and diastolic blood pressures (SBP and DBP, respectively) in G6PD^N126D^ than G6PD^S188F^ of WT rats. *D*, *E*, expression of *Serpine1* and *Abra* was increased in aortas from G6PD^N126D^ but not G6PD^S188F^ rats after Ang II infusion. *F-I*, expression of *Trib1*, *Npr3*, *Myocd* and *Myh11* was increased in aortas from G6PD^S188F^ but not G6PD^N126D^ rats after Ang II infusion. N = 8 to 10/group in *A*-*C* and n = 5 to 6/group in *F*-*I*. Not significant (ns), ∗*p* < 0.05; ∗∗*p* < 0.005; ∗∗∗*p* < 0.001, and ∗∗∗∗*p* < 0.0005.

Because we found differences in metabolism, DNA methylation and gene expression, and aortic stiffness and blood pressure between G6PD^N126D^ and G6PD^S188F^ rats, we tested whether the G6PD^N126D^ rats develop more severe vascular diseases than G6PD^S188F^ or WT rats. To do so, we used a well-established model in which chronic Ang II infusion leads to the development of vascular fibrosis, hypertension, and heart failure (44, 45). Ang II (400 ng/kg/min) infusion for 14 days increased (*p* < 0.001) aortic stiffness and blood pressure in WT rats, but those effects were 45 to 50% (*p* < 0.0001) greater in G6PD^N126D^ than WT or G6PD^S188F^ rats (Fig. 12, *A*–*C*). In addition, Ang II induced greater (*p* < 0.05) expression of genes encoding SERPINE1 (Fig. 12*D*) and ABRA (Fig. 12*E*) in aortas from G6PD^N126D^ than G6PD^S188F^ or WT rats. By contrast, expression of genes encoding TRIB1 and NRP3 was stronger in aortas from G6PD^S188F^ than G6PD^N126D^ rats. Moreover, in the Ang II infusion model, expression of MYOCD and MYH11 was increased in aortas from G6PD^S188F^ rats but not G6PD^N126D^ rats (Fig. 12, *F*–*I*). These results suggest that the G6PD^N126D^ genotype is more likely to activate transcription of genes that encode maladaptive proteins and increase the severity of chronic Ang II-induced vascular diseases than the G6PD^S188F^ or WT genotype.

## Discussion

The results of this study demonstrate that in vascular tissue modifications of the 3D organization of the genome control expression of *Tet* genes encoding DNA demethylases, which remove DNA methylation marks and turn on gene expression. The 3D organization of the genome is now considered to be a crucial determinant of the transcriptomic makeup of a cell. G6PD is a critical enzyme in a branch of the glycolytic pathway. Among the numerous known G6PD polymorphisms, the deficient S188F polymorphism was first detected in individuals from the Mediterranean region, while the non-deficient N126D polymorphism was originally found in individuals from sub-Saharan Africa. Our results demonstrate that introducing the G6PD^S188F^ variant but not the G6PD^N126D^ variant into rats through CRISPR-editing modifies the 3D organization of the genome, leading to the formation of new chromatin loops in all three *Tet* gene segments and increases their expression in the aorta. Inhibition of TET activity decreased aortic expression of *Myocd, Trib1,* and *Klf13*. This suggests TET2 enzyme, which reportedly promotes expression of the SMC phenotypic program (46), contributes to the transcription of genes encoding MYOCD, a SMC-restricted protein that prevents SMC dedifferentiation (40), and TRIB1, a pseudokinase that prevents SMC proliferation (41). Additionally, our finding that a selective inhibitor of G6PD activity decreased aortic stiffness and blood pressure in WT mice but not TET2 knockout mice suggests that increased *Tet2* expression potentially contributes to the reduction in aortic stiffness seen in rats carrying the G6PD^S188F^ variant.

Previous studies from our laboratory revealed that inhibiting G6PD activity increases *Tet2* expression and reduces pulmonary artery remodeling (34). In addition, we showed that TET-dependent DNA hypomethylation activates *Klf13*, and its product, KLF13, mediates suppression of TGFβ-targeted genes, including *Serpine1* and *Col1a* (21), thereby suppressing expression of fibrotic proteins in aortas from G6PD^S188F^ rats. By contrast, DNA methylation repressed *Klf13* in aortas from G6PD^N126D^ rats and enhanced expression of *Serpine1*, encoding pro-thrombotic PAI-1, in aortas from untreated and Ang II-treated G6PD^N126D^ rats. In that regard, rats carrying the G6PD^N126D^ variant develop severe VEGFR blocker-induced pulmonary hypertension with pulmonary thrombosis (47), and case reports suggest individuals carrying the African G6PD^N126D^ variant are likely at higher risk of developing deep vein thrombosis and pulmonary emboli with right heart failure (48, 49). In the present study, we detected a heretofore unappreciated function of PAI-1 in rats, which we showed to increase aortic stiffness *ex vivo* and to be associated with fibrosis or ECM remodeling and aortic stiffness *in vivo*. In that context, a recent study showed that PAI-1 inhibition leads to cytoskeletal reorganization and reduction of the intrinsic stiffness of SMCs *ex vivo* (50). In addition to demonstrating that the G6PD^N126D^ variant elicits expression of genes encoding proteins detrimental to vascular function, our results also show that arterial stiffness and blood pressure are significantly higher in untreated and Ang II-treated G6PD^N126D^ rats than in WT or G6PD^S188F^ rats. Thus, our findings show that two polymorphisms in the same protein evoke distinct modifications of the 3D organization of the genome, transcriptomic profile, and phenotype of cells in vascular tissue, and as a consequence contribute to different outcomes in rats with Ang II-induced vascular disease. These findings have direct clinical implications because epidemiological studies have shown that while individuals carrying the G6PD^S188F^ variant exhibit protection from vascular disease and cancer (12, 51), those carrying the G6PD^N126D^ variant exhibit greater susceptibility to those diseases (13, 52, 53). Additionally, because G6PD is X-linked and X inactivation is impaired with aging, males exhibit different G6PD expression/activity than females with aging. Therefore, the findings reported here are also relevant to individuals not carrying the G6PD^S188F^ or G6PD^N126D^ variant.

DNA sequences and the 3D organization of the genome (chromosomal interactions) are now thought to be factors underlying gene function and interactions between distant fragments, which may regulate transactivation activity *via* mediator proteins (4). Recent studies using chromatin conformation capture technologies have mapped genome-wide chromatin interactions and have demonstrated the importance of interacting domains, such as TADs and chromatin loops, to transcriptional regulation of genes in cancer cells (5, 6). TADs and chromatin loops change during the development of an organism, aging and disease, and exhibit cell- and condition-specific differences (7). One study investigating the biological significance of various boundary changes found that genes around merged and non-differential TAD boundaries are enriched in a variety of developmental and differentiation processes, while strength change TAD boundaries were not enriched in any biological processes (7). Our present findings indicate that modifications of the 3D genomic organization are critical regulators of gene expression, including the genes encoding the DNA demethylases that remove DNA methylation marks in arterial cells, thereby affecting vascular structure and function. Other studies suggest epigenetic modifications may play a key role in vasculopathies (54, 55). Given that DNA methylation reportedly modifies the 3D genomic organization, increases chromatin condensation in pericentromeric regions, favors the heterochromatin state, and decreases DNA flexibility (25), we suggest that the DNA hypomethylation observed in aortas from G6PD^S188F^ rats contributes to new TAD boundary and chromatin loop formation around genes throughout the whole genome. This includes around the *Tet* segments in vascular cells. Our results further suggest the greater H3K27ac enrichment seen in G6PD^S188F^ rats suggests G6PD deficiency leads to increased histone acetylation, which contributes to the conformational modification of the 3D genome and loop formation around *Tet* genes, increasing their expression. Consistent with that idea, eigenvector results showing the A compartment (B-to-A compartment transition in G6PD^S188F^ rats as compared to the WT or G6PD^N126D^ rats) on chromosome 2 around the *Tet2* coordinates imply a euchromatin structure that allows accessibility to transcription factors and promotion of enhancer-promoter interactions. Notably, the G6PD^S188F^ variant, but not the G6PD^N126D^ variant, modifies TAD boundary architecture and elicits the formation of new chromatin loops around the *Tet2* gene segment and upregulates aortic expression of *Tet2*.

Additionally, our findings suggest that the interaction between G6PD and MATR3, along with the increased relative abundance of MATR3 and CTCF protein, potentially contributed to a modification of the 3D-genome structure leading to the formation of new TAD boundaries and chromatin loops in G6PD^S188F^ rats. MATR3 forms a complex with CTCF and the cohesin complex, leading to widespread effects on 3D genome architecture, whereas loss of MATR3 perturbs CTCF–cohesin occupancy at a subset of genomic loci (27). Although the cohesin subunits SMC1A and SMC3 were expressed at lower levels in G6PD^S188F^ rats than in G6PD^N126D^ rats, SMC3 exhibited distinct post-translational modification. Inhibition of SMC3 deacetylation, as well as knockdown of SMC3 decreased *Tet2* expression in SMCs and aortas from WT and G6PD^S188F^ rats, indicating that chromatin loop formation and higher-order 3D genome organization play a critical role in *Tet2* transcriptional regulation. Consistent with this mechanism, acetylation of SMC3 converts cohesin into a PDS5A-bound state, thereby pausing loop extrusion (32). Notably, loss of SMCHD1—a cohesin-like protein—disrupts long-range intra-chromosomal interactions between B compartments while promoting the formation of new TADs and chromatin loops (30). Inactivation of SMCHD1 also induces widespread B-to-A compartment transitions, accompanied by transcriptional activation of previously silenced genes (30). Consistent with these observations, our findings that SMCHD1 is significantly downregulated in G6PD^S188F^ rats and post-translationally modified in G6PD^N126D^ rats, together with the concomitant B-to-A compartment transition observed in G6PD^S188F^ rats, suggest that SMCHD1 and MATR3 jointly contribute to the establishment of new TAD boundary architecture and chromatin loop formation around *Tet2* and SMC-restricted genes, *Myocd* and *Lmod1*, in vascular tissue.

Further, our findings unequivocally demonstrated that the G6PD^S188F^, but not the G6PD^N126D^, variant evokes unique conformational changes in the 3D genomic organization, leading to the formation of new chromatin loops around genes encoding TETs, NOS3, TRIB1, CACNA1C, MYOCD and LMOD1. All these proteins prevent SMCs from dedifferentiating and preserve their contractile function. We and others have reported that TET2 hypomethylates the promoters of SMC-restricted genes encoding the transcription co-activator MYOCD and other SMC contractile proteins that maintain a differentiated SMC phenotype (34, 35, 46). Moreover, expression of *Trib1* and *Npr3* is driven by DNA hypomethylation (56). Conversely, because new chromatin loops were observed around *Serpine1* in the genome of G6PD^N126D^ rats, we suggest that the expression of this gene is upregulated in the aortas from G6PD^N126D^ rats, reflecting the downregulation of *Klf13*, which suppresses TGFβ signaling elicited expression of genes encoding ECM proteins and PAI-1 (21). Therefore, these results suggest the G6PD^S188F^-mediated modification of the 3D organization of the genome increased TET2 expression and TET2-dependent proteins, including SMC-restricted proteins.

G6PD expression and activity are increased in coronary artery disease and pulmonary hypertension (34, 57, 58, 59). In addition, we and others previously showed that increased G6PD contributes to the pathogenesis of vascular remodeling (34, 35, 60, 61, 62) and that application of a G6PD inhibitor reduces aortic stiffening and blood pressure (16, 34, 35, 61). Interestingly, in individuals carrying the G6PD^S188F^ variant, there is no change in the protein’s expression and no shift in its electrophoretic mobility (11). However, there is a >80% loss of G6PD enzyme activity, and these individuals are less susceptible to cardio- and cerebrovascular disease (12). We also recently showed that the G6PD^S188F^ variant reduces hypertension induced by a short-term (12 months) high-fat diet feeding or Ang-II (63, 64) and improves cardiac function and exercise tolerance (65). By contrast, in individuals carrying the G6PD^N126D^ variant, there is no change in the protein’s expression, an increase its electrophoretic mobility and a 10 to 20% loss of enzymatic activity (11), and these individuals are more susceptible to vascular disease and heart failure (13). Consistent with those characteristics, we found that Ang-II elicits greater hypertension and aortic stiffness in G6PD^N126D^ than WT rats. However, there are conflicting reports regarding the role of G6PD deficiency in the pathogenesis of vascular diseases (12, 13, 51, 52, 53, 58, 66, 67, 68, 69). The discrepancies in the literature are mainly because retrospective epidemiological studies are confounded with other non-vascular diseases and are not well controlled (63). Moreover, epistasis and differences in the epigenetics of individuals also may contribute to the observed mixed effects of G6PD deficiency on cardiovascular pathology. Our genetically engineered rat models faithfully mimic the human G6PD deficiency phenotype with no confounding variables. This makes our present findings the first experimental evidence showing that the deficient Mediterranean and non-deficient African G6PD variants have opposite effects on the vasculature, which corroborates the epidemiological studies indicating that individuals carrying the Mediterranean but not the African G6PD variant are less susceptible to vascular disease (12, 51, 69). We therefore suggest that a nonsynonymous single-nucleotide polymorphism at two different positions in G6PD could be the basis for the controversial effects of G6PD mutations on the diverse pathological outcomes of vascular diseases observed in different ethnic groups.

Worldwide, over 500 million individuals (6% of humanity) carry a loss-of-function G6PD mutation (38). Clinically, this manifests as acute neonatal jaundice, acute hemolysis following exposure to oxidative stress, or severe chronic non-spherocytic hemolytic anemia (70). However, the contribution of G6PD deficiency to cardiovascular function has rarely been studied. It was therefore of paramount importance to determine the molecular mechanism(s) behind the disparate effects of the G6PD^S188F^ and G6PD^N126D^ variants on vascular diseases. Metabolic reprogramming is now considered a critical cause and driver of many diseases, including vascular disease. The G6PD is a key enzyme in the PPP, a branch of glycolytic pathway, that produces cell’s reducing power and five carbon ribose sugar required for the *de novo* synthesis of RNA and DNA. While the loss of G6PD function is expected to increase oxidative stress, we paradoxically found that the non-deficient G6PD^N126D^, but not deficient G6PD^S188F^, variant increased reductive stress (elevated GSH-to-GSSG ratio). Additionally, the G6PD^N126D^ variant reportedly increases levels of the TCA cycle metabolite, glycerophospholipids shingosine-1-phosphate and phosphatidylethanolamine, which are known to modify vascular function and contribute to vascular remodeling (71, 72, 73, 74), as well as tryptophan metabolites, which are associated with vascular inflammation and atherosclerosis (75, 76). Intriguingly, the G6PD^N126D^ variant decreased 6-ketoglutarate, which is a co-factor for TET enzymes (77), and the 6-ketoglutarate-to-2-hydroxyglutarate ratio in the aorta. By contrast, 2-hydroxyglutarate, which inhibits TET enzymes (77), was decreased in aortas from G6PD^S188F^ rats. We therefore propose that metabolic reprogramming and differential 3D genomic organization associated with the Mediterranean and African G6PD polymorphisms could be fundamental to differential gene expression potentially responsible for the disparate outcome of vascular diseases in individuals from Mediterranean and African origin. This also raises a broader question regarding the involvement of other proteins and polymorphisms in regulating the 3D organization of the genome that may affect vascular function and structure, which should be investigated in the future.

A healthy aorta exerts a powerful cushioning effect that limits arterial pulsatility and protects the microvasculature from potentially harmful fluctuations in pressure and blood flow (78). Aortic (large artery) stiffness or loss of Windkessel function, which increases with aging and connective tissue diseases, impairs this cushioning effect and, consequently, increases the risk of hypertension and damage in organs such as the heart and kidneys (78, 79, 80). Further, aortic stiffening is accelerated by obesity or a high-fat diet (81) and by elevated Ang II levels (80). Interestingly, aging increases collagen and advanced glycation end products (81). Vascular calcification, SMC senescence, increased oxidative stress, loss of nitric oxide and inflammation all contribute to ECM remodeling and aortic stiffening (79). SMCs, endothelial cells, and fibroblasts all play key roles in the pathogenesis of aortic stiffness (80). Thus, aortic stiffness is a multifactorial condition. It is a silent killer and there is as yet no adequate treatment available to reduce aortic stiffness and the associated ECM remodeling. Based on our findings, we propose a new paradigm wherein the nexus between deficient but not non-deficient G6PD variant-dependent alteration of metabolism and MATR3 expression-modified 3D genomic organization potentially prevents the expression of maladaptive genes and the development of aortic stiffness and vascular disease.

### Limitation

In this study, genome-wide DNA methylation was assessed using WGBS, which does not distinguish between 5-methylcytosine (5 mC) and 5-hydroxymethylcytosine (5 hmC), as both are retained as cytosine after conversion. To address this limitation, locus-specific analysis of hydroxymethylation (*e.g.*, hMeDIP-PCR) at *Klf13* and *Serpine1* will be necessary to define the contribution of TET-mediated conversion of 5 mC to 5 hmC in transcriptional regulation. Additionally, elucidating the structure-function relationship of the G6PD-MATR3 interaction will be essential to determine how this axis regulates 3D genome organization and downstream gene expression, thereby providing deeper mechanistic insight into metabolic control of transcription.

## Experimental procedures

### Animal Models and experimental protocols

All animal protocols [Protocol#: A3362-01] were approved by the New York Medical College, Institutional Animal Care and Use Committee. All procedures followed the guidelines of the NIH Guide for the Care and Use of Laboratory Animals. The Medical College of Wisconsin and the University of Michigan Transgenic Animal Model Core developed the rat models used in this study. Briefly, a Sprague-Dawley rat model harboring a S188F or N126D substitution in the coding region of *G6pd* was developed using a 3-component CRISPR editing approach and on- and off-target effects were determined by Sanger sequencing and were reported previously (47, 63, 82). As *G6pd* is an X-linked gene, age-matched male [350–450 g] rats with the G6PD^S188F^ or G6PD^N126D^ variant or WT G6PD were used to perform all studies. Rats were kept in 12/12 h light/dark cycle and had full access to food (normal chow diet) and water. Rats were anesthetized with 2% isoflurane. After hemodynamic measurements, rats were euthanized with Euthasol (1 ml intravenous bolus) and the aorta and lungs were harvested enbloc for biochemical analysis. Arteries were also fixed in formalin to generate paraffin blocks for histological analysis. This study was performed in a randomized and double-blinded manner.

### Echocardiography

Echocardiography was performed using a Vevo 770 imaging system (VisualSonics, Toronto, ON, Canada). During the echocardiography, body temperature of the rat was maintained using a heating pad. Briefly, pulse wave velocity (PWV) was determined from transit time between Doppler flow signals in the carotid and iliac arteries. The pulse-transit time from the carotid to iliac arteries (T) was calculated by subtracting the R-carotid foot time interval from the mean R-iliac foot time interval. The distance (D) between the points of probe applanation over the carotid and iliac arteries was measured using a tape measure. PWV was calculated as: PWV = Distance (D)/Time (T). The carotid peak velocity was measured with pulsed wave Doppler.

### Hemodynamics

Rats were anesthetized using 2% isoflurane was used to maintain anesthesia during the entire duration of the surgery and data acquisition. During the hemodynamic measurements, body temperature of the rat was maintained using a heating pad. About 3 cm^2^ of skin over the ventral neck region was exposed to locate the right common carotid artery and a 2.0 F Millar Micro-Tip pressure catheter was inserted into the right common carotid artery, and systolic blood pressure was measured. The MPVS Ultra Single Segment Pressure-Volume Unit catheter (Millar Instruments). Data were acquired and analyzed using PowerLab (ADI Instruments).

### H&E staining of aortic cross section

Aorta fixed in 10% NBF was blocked in paraffin, after which 5-μm sections were cut and stained with hematoxylin/eosin and Masson’s Trichrome. Sections were examined in a blinded manner under the microscope (Olympus BX40, Olympus America) at 200X magnification.

### Atomic Force Microscopy

Atomic force microscopy [AFM] was used to measure the stiffness of isolated rat aortas as previously described (83). For analysis, small sections (approximately 2 × 4 mm) of the descending aorta were excised using microsurgical scissors, affixed to a 35-mm culture dishes with adhesive, and immersed in 4 ml of PBS. AFM in contact mode was applied to each aorta using an NX12 AFM [Park Systems] mounted on a Nikon ECLIPSE Ti2 inverted microscope. The aorta was indented using a three-sided pyramidal tip (8 nm in radius) against a silicon nitride cantilever [spring constant = 0.059 N/m; BL-AC40TS-C2, Oxford Instruments]. Approximately 10 force-distance curves were collected from three separate locations within each aorta. The force-distance curves from each indentation were saved as tiff files and uploaded to XEI software [Park Systems] for analysis. Stiffness [elastic modulus] was determined by fitting the first 400 nm of horizontal tip deflection to the Hertz model.

### RNAscope and microscopy

RNAscope was performed with FFPE slides containing rat aorta as previously described (84). Briefly, the specimens were fixed in 10% paraformaldehyde for 24 h before being processed as instructed in the RNAscope Multiplex Fluorescent Reagent Kit v2 Assay user manual provided by ACD. Slides were baked in a dry oven for 1 h at 60 °C before undergoing deparaffinization, which entailed two 5-min washes in xylene with slight agitation, followed by two 2-min washes in 100% ethanol with slight agitation. Slides were then dried in a drying oven at 60 °C for 5 min. Five drops of RNAscope H_2_O_2_ were applied to each tissue section and spread so the tissue was fully covered, after which the sections were incubated at room temperature for 10 min and then washed twice in deionized [DI] water. An Oster Steamer was filled with tap water to the “Max Fill” line. After heating the water to 99 °C (checked with a scanning thermometer), containers filled with DI water and RNAscope 1× Target Retrieval Reagent were placed in the steamer. Slides were acclimated in the DI water for 10 s before being placed in the RNAscope 1× Target Retrieval Reagent for 15 min (considered “mild” treatment). Slides were then removed and washed with DI water for 15 s before being placed in a drying incubator at 60 °C for 5 min. A hydrophobic barrier was drawn around each tissue section and allowed to dry for 5 min before five drops of RNAscope Protease Plus were applied to cover each tissue section, which were then placed in a HybEZ Humidity Control Tray in a HybeEZ oven at 40 °C for 15 min. The slides were then washed twice in DI water. On the day of the procedure, probes were prepared exactly as described in the user manual. Five drops of the probe mixture were applied to each tissue section and spread so the entire section was covered before incubation in the HybEZ Humidity Control Tray in a HybeEZ oven at 40 °C for 2 h. The slides were then washed with 1× Wash Buffer and left in 5× SSC Buffer overnight at room temperature. The following morning, the slides were washed twice with 1× Wash Buffer for 2 min at room temperature. Because we used three probes, three Hybridization AMP steps were performed as instructed in the user manual before the HRP C1 [Opal 690] and HRP C2/C3 [Opal 570] signals were developed. Lastly, the slides were mounted and counterstained with DAPI using SouthernBiotech© DAPI Fluoromount-G [Catalogue #: 0100-20] and allowed to dry overnight at room temperature in the dark. The following day, the slides were imaged on a Zeiss LSM 980 + Airyscan 2 High-resolution confocal microscope, after which the images were processed using Zen Blue 3.3. Images were obtained under 40× [water-immersed] magnification using two lasers: for Cy3, 561 nm at 1.5% strength and 650 V gain; for Cy5.5, 639 nm at 2.5% strength and 650 V gain.

### Measurement of G6PD activity

G6PD activity was measured spectrophotometrically with an assay purchased from Cayman Chemicals, MO. Five-microliter aliquots (containing 5 μg of protein) of homogenate were pipetted into the wells of a 96-well plate (Fisher Scientific) followed by the addition of 200 μl of the standard assay buffer. The absorbance at 339 nm was then measured using a plate reader (Synergy HT, Biotek) immediately and for up to 25 min at 50-s intervals. Background absorbance was corrected by subtracting the value of a blank containing no homogenate from all sample readings, and G6PD activity was determined quantitatively using a molar extinction coefficient of 6220 M^−1^ cm^−1^.

### RNA expression by quantitative PCR

mRNA analysis was performed by QPCR as previously described (85). Total RNA was extracted from aorta using a Qiagen RNeasy kit (Cat # 217004) and cDNA was prepared using SuperScript IV VILO Master Mix (Cat # 11756500, Invitrogen) for mRNA. Quantitative PCR was performed in triplicate using TaqMan Universal PCR Master mix (Cat #4324018) using a Mx3000p Real-Time PCR System (Stratagene, Santa Clara, CA). mRNA expressions were normalized to internal control *Tuba1a*, and relative mRNA expression was determined using the 2^−ΔCT^ method.

### Bulk RNA sequencing

RNAseq was performed as previously described (47). Briefly, library prep was performed with 300-ng RNA samples isolated from aorta using the ribosomal RNA depletion method. Sequencing of paired end reads was performed using an Illumina NextSeq 550 System (75 bp x2), and single-end sequencing was performed (30 million reads/replicate).

### Silencing of SMC3

To knock down SMC3 in SMCs and aorta, we used antisense oligonucleotides (ASOs), which are single-stranded synthetic nucleic acids that recognize and specifically bind target mRNA *via* Watson–Crick base pairing (86). We employed a next-generation ASO incorporating a 2′-deoxy-2′-fluoro-β-D-arabinose (FANA) sugar modification and a phosphorothioate backbone, consisting of a central DNA segment flanked by FANA-modified nucleotides, directed against SMC3. ASOs (AUM*silence* sdASO; see Table 1) targeting SMC3 were designed and synthesized by AUM BioTech, LLC based on the Rattus norvegicus SMC3 mRNA sequence obtained from NCBI (NM_031583.2). A scrambled FANA ASO was used as a negative control. AUM*silence* sdASOs are self-deliverable antisense oligonucleotides for mRNA knockdown and do not need any transfection reagents or delivery agents for either *in vitro* or *in vivo* applications, and are suitable for use in primary cells (87, 88).

### Metabolomics

Metabolomics analysis was performed as previously described (47, 85, 89, 90). Briefly, the aorta was crushed (SPEX GenoGrinder) and extracted using a mixture containing a 5:3:2 (v/v/v) ratio of cold methanol:acetonitrile:water with 10 mg/ml tissue. The metabolomic was performed using a Vanquish UHPLC system and Q Exactive mass spectrometer (Thermofisher). Thereafter, samples were run over a Kinetex C18 column (2.1 × 150 mm, 1.7 μm Phenomenex) under isocratic conditions (5% acetonitrile, 95% water, 0.1% formic acid, 250 μl/min flow rate). MAVEN was used to assign the metabolites, and multivariate statistical analysis (partial-least-squares discrimination) was used for data normalization.

### Rapid Immunoprecipitation Mass Spectrometry of Endogenous Proteins (RIME) analysis

Rat aortas were excised and placed in cryogenic vials or microcentrifuge tubes, which were immediately submerged in liquid nitrogen for 2 min and then stored at −80 °C. Subsequently, the tissue was pulverized, and RIME was carried out using an antibody against G6PD (Custom Antibody) with 150 μg of chromatin from rat aorta to identify proteins that interact with G6PD and analyzed as described previously (91).

### Mass spectrometry-based proteomics

Quantitative proteomic analysis was performed as previously described (28). Briefly, frozen samples were homogenized in freshly prepared high-salt buffer (50 mM Tris-HCl, 3 M NaCl, 25 mM EDTA, 0.25% w/v CHAPS, pH 7.5) containing 1× protease inhibitor (Halt Protease Inhibitor, Thermo Scientific) at a concentration of 10 mg/ml. After protein extraction, the samples were digested according to the filter-aided sample preparation (FASP) protocol using a 10-kDa molecular weight cutoff filter. Peptides were recovered from the filter using 50 mM AB. Samples were dried in a Speed-Vac and then desalted and concentrated on a Thermo Scientific Pierce C18 Tip. Each sample (20 μl) was loaded onto individual Evotips for desalting, and MS-MS-TOFF was performed. MS/MS spectra were extracted from raw data files and converted into.mgf files using MS Convert (ProteoWizard, Ver. 3.0). Peptide spectral matching was performed with Mascot (Ver. 2.5) against the Uniprot rat database. Scaffold (version 4.8, Proteome Software, Portland, OR, USA) was used to validate MS/MS-based peptide and protein identifications. Peptide identifications were accepted if they could be established at greater than 95.0% probability as specified by the Peptide Prophet algorithm. Protein identifications were accepted if they could be established at greater than 99.0% probability and contained at least two identified unique peptides. The following variables PTM, were determined. The modifications typically included in the workflow are Cysteine modifications: oxidation (+15.9949 Da), dioxidation (+31.9988 Da), dehydroalanine (Dha, −34.0809 Da), glutathionylation (+305.0682 Da), and N-ethylmaleimide (NEM) alkylation (+125.0477 Da); Lysine modifications: GlyGly remnant from ubiquitination (+114.0429 Da) and methylation (+14.0156 Da); Aspartate methylation: (+14.0156 Da); Asparagine and glutamine deamidation: (+0.9840 Da); and N to D methylation: (+14.9997 Da).

### HiC sequencing

To determine 3D genomic structure, HiC was performed with aortas from male and female WT, G6PD^N126D^ and G6PD^S188F^ rats. Briefly, HiC was performed by Arima Genomics. The HiC libraries were sequenced on an Illumina HiSeq2500 sequencer in “paired-end” mode with a 2 × 150 bp read length and a read depth of ∼548 million-1.2 billion reads per sample. This read depth enabled analysis of genomic features requiring various levels of resolution. We analyzed the resulting data using Juicer v1.6 for A/B compartment calls, TADs and chromatin loop calls, which can be visualized using Juicebox (92). We also analyzed the HiC dataset with the modified TADCompare program, an R language package, used for temporal analysis of TADs and for differential analysis of boundaries of interacting domains between two or more HiC datasets modified by our lab (93).

### Whole-Genome Bisulfite Sequencing (WGBS)

Genomic DNA was isolated from ∼5 mg of tissue using the MasterPure DNA Purification Kit according to the manufacturer’s instructions. DNA was resuspended in TE buffer and quantified by fluorometry. Bisulfite conversion was performed on 50 to 100 ng of genomic DNA using the Zymo EZ DNA Methylation Lightning Kit following the manufacturer’s protocol. Bisulfite-treated DNA was purified and used to prepare sequencing libraries with the EpiGnome Kit, which selectively amplifies the complement of the original bisulfite-converted DNA. Libraries were sequenced on an Illumina HiSeq 2500 platform using 75-bp paired-end reads. Raw FASTQ files were quality-filtered and adapter-trimmed using Trimmomatic, and reads were aligned to a bisulfite-converted reference genome using Bismark with Bowtie2. Genome tracks were visualized using IGV. CpG sites with ≥30 × coverage were included for analysis. Differentially methylated loci (DMLs) and regions (DMRs; 1-kb windows) were identified using MethylKit, with statistical testing based on sample duplication (Fisher’s exact test or logistic regression). Results were filtered for *p* < 0.05 and ≥ 25% methylation difference and annotated relative to RefSeq transcription start sites. WGBS was performed by Genewiz.

### Cut and run analysis

Nuclei were isolated using a Chromium Nuclei Isolation kit (10× Genomics, Cat#PN-1000493) per the manufacturer’s protocol. The following antibodies were used to generate the DNA: Abcam rabbit anti-histone H3 acetyl 27 pAb Cat# ab4729 and EpiCypher IgG pAb (Cat# 13-0042). The Cutana ChIC/CUT&RUN (EpiCypher Cat# 14-1048) and CUT&RUN library prep (EpiCypher Cat# 14-1002) kits were purchased, and the manufacturer’s protocol was followed to generate the DNA libraries. A Qubit fluorometer with a 1× dsDNA HS assay kit was used with a Bioanalyzer system high-sensitivity DNA kit to determine the QC for DNA library quality. Finally, for final CUT&RUN sequencing, the libraries were run on the Illumina sequencing platform at the University of Rochester GRC, Rochester, NY. The IGV browser was used to visualize.bigWig and.stringent.bed files, where the IgG controls were used as the inputs for empirical FDR/threshold values for all H3K27ac SEACR samples (94).

### Statistics

Statistical analyses and graphs were generated using GraphPad Prism 10.0 [GraphPad Software, Inc, La Jolla, CA] and Metaboanalyst 3.0 (95). All experiments were done in triplicate [technical replicates] to ensure the reliability of single values. Normality and outlier [Robust Regression and Outlier Removal: ROUT; Q = 1%] identification tests were performed, and outliers were removed. Values are presented as the mean ± SD; n represents the number of rats per group or biological replicates. Comparisons between multiple groups with n > 6 rats were performed using two-way ANOVA followed by Tukey’s multiple comparisons test. For groups with n < 6, comparisons were made using the non-parametric Kruskal-Wallis test followed by Dunn’s multiple comparison test. Comparisons between two groups were made with Student’s *t* test. Values of *p < 0.05* were considered significant.

## Data availability

All data is included in the manuscript, and the data will be available upon request to the corresponding authors.

## Supporting information

Additional results are included in the online supplement.

## Conflict of interest

The authors declare that they have no conflicts of interest with the contents of this article.
