# Supplementary material for: Glucose-6-phosphate dehydrogenase variants modify 3D genomic organization to suppress maladaptive gene expression and vascular disease
Source: J Biol Chem. 2026 May 27;302(8):113204. doi: 10.1016/j.jbc.2026.113204 (PMC13400228; doi:10.1016/j.jbc.2026.113204)
Supplement: Supplemental Data [file mmc1.docx]

**SUPPLEMENTAL DATA**

**Glucose-6-phosphate dehydrogenase variants modify 3D genomic organization to suppress maladaptive gene expression and vascular disease**

Christina Signoretti,^1#^ Shun Matsumura,^1#^ Melinee D’silva,^1^ Comfort Williams,^1^ Brenden Marshall,^1^ Samuel Fatehi,^1^ Rhonda Drewes,^2^ Abby L. Grier,^3^ Monika Dzieciatkowska,^3^ Angelo D’Alessandro,^3^ Yongho Bae,^2^ Sachin A. Gupte^1*^

**SUPPLEMENT Figures**

**Supplement Figure 1: Bulk RNA sequencing of the aorta of wild-type (WT) versus G6PD^N126D^ and G6PD^S188F^ rats.** (A-B, D-E) Volcano plot Log2fold change and corresponding genes depict upregulated (red) and downregulated (blue) genes in the aorta of wild-type versus G6PD^N126D^ and G6PD^S188F^ rats respectively (n=6/group). (C, F) Gene ontology terms for the aorta of G6PD^N126D^ versus wild-type and G6PD^S188F^ versus wild-type rats (n=6/group).

**Supplement Figure 2: Comparison of GO terms of the aorta of G6PD^N126D^ versus G6PD^S188F^ rats.** Gene ontology terms for the aorta of G6PD^N126D^ versus G6PD^S188F^ rats (n=6/group).

**Supplement Figure 3: Localization of *Ccl5* in the aorta of wild-type rats.** RNAscope microscopy displayed *Ccl5* expression in the adventitia/inside of the aorta (DAPI; blue, *Ccl5*; yellow). Representative image (20x magnification) where n=5/group.

**Supplement Figure 4: Principal component graph demonstrating differential DNA methylation between the genotypes.** Whole genome bisulfite sequencing results demonstrate differential DNA methylation between wild-type (red circles; WT-R; right panel) and G6PD^S188F^ (green circles; G6PD^S188F^-G; right panel) rats but not between wild-type (red circles; WT-R; left panel) and G6PD^N126D^ (green circles; G6PD^S188F^-G; left panel)

**Supplement Figure 5: Methylation of *Serpine1* promoter.** CpG island map in rat *Serpine1* gene promoter is shown (Top panel). CpG island in middle and proximal region of *Serpine1* gene is methylated in G6PD^S188F^ rats as compared to wild-type (WT) rats (Bottom Panel).

**Supplement Figure 6: Changes in the 3D genome on chromosome 4 where *Nos3* and *Cacna1c* genes are located.** Representative .hic files zoomed into 25KB (top) and 1KB (bottom) resolution for chromosome 4 using Juicebox visualization tool. 2D annotations analysis of *Nos3* location of interest showed chromatin loops for both G6PD^N126D^ and G6PD^S188F^ (small blue squares) as well as chromatin loops for wild-type control (small green squares). 2D annotations analysis of *Cacna1c* location of interest showed chromatin loops for both G6PD^N126D^ and G6PD^S188F^ (small blue squares) as well as chromatin loops for wild-type control (small green squares) and TADs (large purple squares). The color range is 0-14 for all images. Merged comparisons include WT & G6PD^S188F^ and WT & G6PD^N126D^ (n=2/group).

**Supplement Figure 7: Changes in the 3D genome on chromosome 7 where *Trib1* gene is located.** Representative .hic files zoomed into 25 KB (top) and 1KB (bottom) resolution for chromosome 7 using Juicebox visualization tool. 2D annotations analysis of *Trib1* location of interest showed chromatin loops for both G6PD^N126D^ and G6PD^S188F^ (small blue squares) and TADs (large black box) for G6PD^S188F^ as well as chromatin loops for wild-type control (small green squares) (n=2/group).

**Supplement Figure 8: Changes in the 3D genome on chromosome 10 where *Myocd* gene is located.** Representative .hic files zoomed into 25 KB (top) and 1KB (bottom) resolution for chromosome 10 using Juicebox visualization tool. 2D annotations analysis of *Myocd* location of interest showed chromatin loops for both G6PD^N126D^ and G6PD^S188F^ (small blue squares) as well as chromatin loops for wild-type control (small green squares) (n=2/group).

**Supplement Figure 9: Changes in the 3D genome on chromosome 13 where *Lmod1* gene is located.** Representative .hic files zoomed into 25 KB (top) and 1KB (bottom) resolution for chromosome 13 using Juicebox visualization tool. 2D annotations analysis of *Lmod1* location of interest showed chromatin loops for G6PD^S188F^ (small blue squares) as well as chromatin loops for wild-type control (small green squares) (n=2/group).

**Supplement Figure 10: Changes in the 3D genome on chromosome 12 where *Serpine1* gene is located.** Representative .hic files zoomed into 25 KB (top) and 1KB (bottom) resolution for chromosome 12 using Juicebox visualization tool. 2D annotations analysis of *Serpine1* location of interest showed chromatin loops for both G6PD^N126D^ and G6PD^S188F^ (small blue squares) as well as chromatin loops for wild-type control (small green squares) (n=2/group).

**Supplement Figure 11: Changes in the 3D genome on chromosome X where *G6pd* gene is located.** Representative .hic files zoomed into 25 KB (top) and 1KB (bottom) resolution for chromosome X using Juicebox visualization tool. 2D annotations analysis of *G6pd* location of interest showed chromatin loops for both G6PD^N126D^ and G6PD^S188F^ (small blue squares) and TADs for G6PD^S188F^ (large black square) as well as chromatin loops for wild-type control (small green squares) and TADs (large purple square) (n=2/group).

**Supplement Figure 12: Blood pressure is decreased by G6PD inhibitor in wild-type mice but not in Tet2^-/-^ mice.** Systolic (SBP) and diastolic (DBP) blood pressure is decreased by N-ethyl-N-[(3 ,5)-17-oxoandrostan-3-yl]urea (1.5 mg/kg/day; sc), a novel G6PD inhibitor (G6PDi), injected daily for 3 weeks in wild-type (Top Panels) but not in *Tet2^-/-^* (Bottom Panels) mice.
